# Heterogeneous-Driven Glutathione Oxidation: Defining the Catalytic Role of Chalcopyrite Nanoparticles

**DOI:** 10.1021/acs.jpcc.3c00987

**Published:** 2023-07-12

**Authors:** Leticia Sanchez-Uriel, Javier Bonet-Aleta, Alfonso Ibarra, Jose L. Hueso

**Affiliations:** † Instituto de Nanociencia y Materiales de Aragon (INMA) CSIC-Universidad de Zaragoza, Campus Rio Ebro, Edificio I + D, C/ Poeta Mariano Esquillor, S/N, 50018 Zaragoza, Spain; ‡ Networking Res. Center in Biomaterials, Bioengineering and Nanomedicine (CIBER-BBN), Instituto de Salud Carlos III, 28029 Madrid, Spain; § Department of Chemical and Environmental Engineering, University of Zaragoza, Campus Rio Ebro, C/María de Luna, 3, 50018 Zaragoza, Spain; ∥ Laboratorio de Microscopias Avanzadas (LMA), Universidad de Zaragoza, Zaragoza 50018, Spain; ⊥ Instituto de Investigación Sanitaria (IIS) de Aragón, Avenida San Juan Bosco, 13, 50009 Zaragoza, Spain

## Abstract

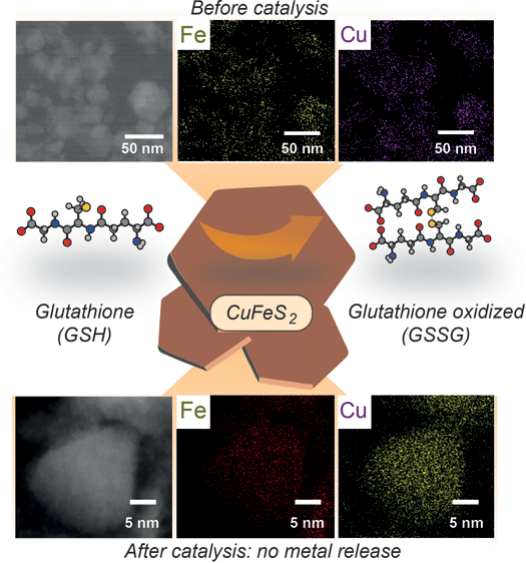

Transition-metal nanocatalysis represents a novel alternative currently experiencing flourishing progress to tackle the tumor microenvironment (TME) in cancer therapy. These nanomaterials aim at attacking tumor cells using the intrinsic selectivity of inorganic catalysts. In addition, special attention to tune and control the release of these transition metals is also required. Understanding the chemical reactions behind the catalytic action of the transition-metal nanocatalysts and preventing potential undesired side reactions caused by acute cytotoxicity of the released ionic species represent another important field of research. Specifically, copper-based oxides may suffer from acute leaching that potentially may induce toxicity not only to target cancer cells but also to nearby cells and tissues. In this work, we propose the synthesis of chalcopyrite (CuFeS_2_) nanostructures capable of triggering two key reactions for an effective chemodynamic therapy (CDT) in the heterogeneous phase: (i) glutathione (GSH) oxidation and (ii) oxidation of organic substrates using H_2_O_2_, with negligible leaching of metals under TME-like conditions. This represents an appealing alternative toward the development of safer copper–iron-based nanocatalytic materials with an active catalytic response without incurring leaching side phenomena.

## Introduction

**Figure 1:**
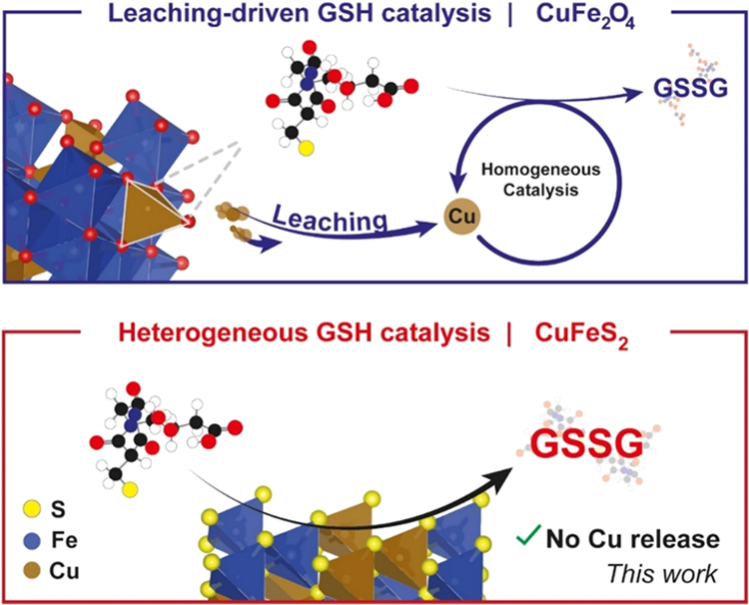
Schematic display of the GSH oxidation steps occurring in the presence of a copper–iron nanocatalyst containing either a spinel-like oxide configuration (top) or a chalcopyrite composition (bottom). For oxide-based catalysts, GSH promotes the Cu release from the crystalline structure through complex formation, and once in solution, GSH is oxidized by the Cu(II)/O_2_ system.^12^ In this work, we present a CuFeS_2_ catalyst that maintains the catalytic activity while preventing the rapid release of Cu ions.

Chemodynamic therapy (CDT) has emerged as a promising alternative to traditional approaches such as radiotherapy (RT), chemotherapy (CT), or surgery given the unique chemical properties of the tumor microenvironment (TME).^1^ High glutathione (GSH)^2^ and hydrogen peroxide (H_2_O_2_) levels,^3,4^ mildly acidic pH,^5^ or relatively low O_2_ concentration^6^ are some chemical features of cancer cells that can be leveraged by nanostructured catalysts to selectively induce cell death. Up to now, the most explored nanocatalysts are based on noble metals such as gold or platinum.^7^ Alternatively, transition-metal oxides containing copper, molybdenum, manganese, and/or iron are also being explored.^7−9^ Several of these oxides are able to trigger a cascade reaction in the presence of GSH and H_2_O_2_.^2^ In a first stage, the metal (M^*n*+^) can react with two molecules of GSH to yield GSSG (Figure 1). Then, the reduced metal (M^(*n*–1)+^), can further convert H_2_O_2_ into hydroxyl radical ^•^OH species through Fenton reactions in a second step.^10^ Overall, the introduction of a transition-metal nanocatalyst can simultaneously deplete antioxidant molecules such as GSH and increase the concentration levels of highly reactive oxygen species (ROS), thereby modifying the redox homeostasis of cancer cells, which are particularly sensitive to this disruption.^11^ Specifically, the combination of Cu and Fe in a single nanoplatform has been demonstrated to be an efficient strategy in cancer therapy.^2,12−15^ The origin of this synergy relies on the role of Cu as a cocatalyst in Fe-driven Fenton reactions.^15,16^ Cu^+^ can react with Fe^3+^ in a thermodynamically favorable process (Δ*E* = 0.6 V) to regenerate active Fe^2+^ species, thus avoiding the highly energetic H_2_O_2_ reaction with Fe^3+^ to yield the desired Fe^2+^ reactive species in the absence of Cu^+^. However, Cu present in Cu–Fe oxides can suffer from lixiviation phenomena under TME conditions^12^ in a similar way as reported for CuO nanoparticles^17^ (Figure 1). Once released into its ionic form in solution, Cu can catalyze GSH oxidation following a homogeneous pathway.^12^ Simultaneously, the remaining Fe-enriched oxides may work as a regenerator of the required O_2_ to maintain the GSH oxidation cycle.^12^ Despite its high catalytic activity, the lack of control in the Cu release can be detrimental to neighboring healthy cells and tissues.^17^

To explore other catalytic pathways that do not entail a huge metal release, we have developed a new synthesis route to chalcopyrite CuFeS_2_ nanoparticles (NPs) and evaluated their catalytic response toward GSH oxidation and H_2_O_2_ conversion into ROS. In our previous research, we identified the in situ formation of Cu–SG complexes when CuFe-oxide NPs reacted with GSH.^12^ Inspired by the chemical strength of the Cu–S bonding in the complex, we have designed these chalcopyrite NPs where Cu occupies tetrahedral sites surrounded by S anions. This reduces the possibility of a direct complexation with GSH and retains Cu within the crystalline network of the NPs avoiding a potential loss of the metal in the human body while maintaining the catalytic activity for both GSH oxidation and ROS production (Figure 1). This work represents an example of a nanocatalyst with a similar reactivity pattern in comparison with an oxide analogue, CuFe_2_O_4_, but with a completely distinct behavior regarding the stability during the reaction, adding valuable alternatives to the catalysts toolbox applied for nanocatalytic therapy.

## Experimental Section

### Chemicals and Materials

Iron(III) chloride hexahydrate (FeCl_3_·6H_2_O, 97%), copper(II) chloride dihydrate (CuCl_2_·2H_2_O, <99%), sodium acetate anhydrous (CH_3_COONa), poly(vinylpyrrolidone) (PVP K30, *M*_W_ 4000 Da), ethylene glycol (EG) (99.8%), sulfur powder (S, ≥99%), poly(ethylene glycol) (PEG, wt 8000) (99.8%), ethanol (CH_3_CH_2_OH, 96%), glutathione (GSH, <98%), glutathione disulfide (GSSG, <98%), ethylenediaminetetraacetic acid disodium salt dihydrate (EDTA 99%), tris(hydroxymethyl)aminomethane (TRIS, ≥99.8%), 3,3′,5,5′-tetramethylbenzidine (TMB, ≥98%), hydrogen peroxide (H_2_O_2_, 33% v/v), dimethyl sulfoxide (DMSO, ≥99.9%), sodium bicarbonate (NaHCO_3_, 99%), hydrochloric acid (HCl, 37%), nitric acid (HNO_3_, 65%), acetonitrile (anhydrous, ACN, ≥99.9%), dimercaptosuccinic acid (DMSA, 99.0%), and phosphate-buffered saline solution (PBS) were purchased from Sigma-Aldrich. Deionized water was obtained from a Milli-Q Advantage A10 System with a resistivity of 18.2 MΩ·cm (Merk Millipore, Germany). All chemicals were used without any further purification.

### Characterization Techniques

The morphology, size distribution, and crystal structure of nanocatalysts were determined by transmission electron microscopy (TEM) on an FEI TECNAI T20 system (Tecnai, Eindhoven, the Netherlands) operated at 200 kV. High-resolution transmission electron microscopy (HRTEM) images were obtained in an image-corrected Titan (FEI) at a working voltage of 300 kV and coupled with a charge-coupled device (CCD) camera (Gatan). The fast Fourier transform (FFT) of several high-resolution TEM images was also analyzed in order to determine the crystalline structure of the samples. High-angle annular dark-field scanning transmission electron microscopy (HAADF-STEM) images were obtained in a Cs-probe-corrected Titan (Thermo Fisher Scientific, formerly FEI) at a working voltage of 300 kV, coupled with a HAADF detector (Fischione). In this mode, the intensity of the signal is proportional to the square of the atomic number (*Z*^2^); therefore heavier elements appear with a much brighter contrast than lighter elements, such as carbon or silicon. It is especially useful to localize metals in organic matrixes. Also, in order to analyze the chemical composition of the materials, X-ray energy-dispersive spectra (EDS) were obtained with an Ultim Max detector (Oxford Instruments).

TEM samples were prepared by resuspension in deionized (DI) water, mild sonication for 30 s, and subsequent drop-casting deposition of 5 μL added onto a holey carbon nickel grid (Electron Microscopy Sciences, Hatfield, PA). Precision tweezers were used to hold the grid and let the droplet dry at room temperature. The hydrodynamic size was determined by dynamic light scattering (DLS) spectroscopy on a Brookhaven 90 Plus instrument (Brookhaven Instruments Corporation, Holtsville, New York). The crystallographic structure was determined by X-ray diffraction (XRD), using Cu Kα radiation and equipment with a PIXcel1D detector (PANanaytical Empyrean). Scan conditions were as follows: 15–100° range and 0.0131° step size. The spectroscopic identification of functional groups was carried out by Fourier transform infrared (FTIR) spectroscopy (Bruker Vertex 70) and by Raman spectroscopy (alpha300 R, Raman Imaging Microscope, WITec, Germany). The experimental conditions were as follows: laser excitation wavelength at 532 nm, 1 mW power, 10 s exposure time, and 3 accumulations. The elemental composition and oxidation states of the elements on the surface were determined by X-ray emitted photoelectron spectroscopy (XPS) (AXIS Supra (Kratos Tech., Manchester, U.K.)) using a monochromatic Al Kα source (1486.6 eV) at 15 kV and 15 mA. Cu and Fe contents were measured on a 4100 microwave plasma-atomic emission spectrometer (MP-AES) instrument (Agilent, Madrid, Spain). Ultraviolet–visible (UV–vis) spectra were recorded using a UV–vis–NIR spectrophotometer (UV-2600i, Shimadzu, Japan). Fourier transform infrared spectroscopy (FTIR) was performed on a Bruker Vertex 70. Metal concentration was determined using Agilent 4100 MP-AES. Mass spectra were collected on a Waters ACQUITY HClass system coupled to a single quadrupole mass spectrometer with an electrospray ionization (ESI) ACQUITY QDa mass detector. Data acquisition and processing were performed by using MASSLYNX software (Waters Co.). ^1^H spectra (D_2_O) were recorded at 25 °C using a Bruker Avance 400 MHz NMR spectrometer and deuterated water as the solvent in a 5 mm QNP probe.

### Synthesis of the Chalcopyrite CuFeS_2_ Nanoparticles

In a typical synthesis, 1.4 g of PVP, 0.2 mmol of FeCl_3_·6H_2_O, and 0.15 mmol of CuCl_2_·2H_2_O were sequentially dissolved in 30 mL of EG under vigorous magnetic stirring, followed by the addition of 44 mmol of CH_3_COONa and 3 mmol of elemental S (scheme displayed in Figure S1). PVP was used as a capping agent to ensure size homogeneity^18^ and EG was used as solvent to prevent agglomeration between particles formed at nearby nucleation points.^2^ The process was carried out in an ultrasonication bath accompanied by N_2_ bubbling to remove O_2_. After stirring for 2 h to ensure the dissolution of metals and potential aggregates, the suspension turned from light green to dark green color. The mixture was placed in a stainless-steel Teflon autoclave and heated at 200 °C for 24 h. After this period, the autoclave was cooled in a cold water bath. Then, several purification and washing cycles were carried out to remove unreacted chemical byproducts. The solid was collected by centrifugation (10,000 rpm for 10 min) several times and washed thoroughly using sequentially ethanol, H_2_O/ethanol mixture (1:1), and H_2_O and 3 mg mL^–1^ PEG in ethanol solution. After stirring for 1 h to ensure surface stabilization with PEG, the CuFeS_2_ NPs were collected again by centrifugation and resuspended in DMSO. The NPs were stored at 4 °C until further use. NP concentration was determined by measuring metal concentration by MP-AES.

### GSH Oxidation Catalysis Monitored by UPLC-MS

The initial GSH concentration was fixed at 5 mM in order to mimic an intracellular ambient.^19^ In a total volume of 5 mL, the catalyst concentration and temperature were adjusted to [Cu] = 10 mg L^–1^ and 37 °C, respectively. pH was fixed at 7.4 using 50 mM TRIS buffer. Sample preparation consisted in diluting a 100 μL aliquot of the reaction into 900 μL of 50 mM TRIS. Experiments involving EDTA were performed by fixing its concentration at 5 mM. For control experiments using CuCl_2_, the concentrations were fixed up to 0.032 and 0.298 mM to test the catalytic activity of the equivalent amount of released Cu for CuFeS_2_ and CuFe_2_O_4_, respectively.

All samples were filtered using 0.22 μm nylon filters before injection in the ultra-performance liquid chromatography-mass spectrometry (UPLC-MS) system. The mobile phase of the UPLC system consisted of an isocratic flow of 0.5 mL min^–1^ of an acetonitrile/water 90:10 mixture without modifiers. The column temperature was set up to 85 °C. The cone voltage of ESI was fixed to 2 V. All intensities obtained of MS spectra were normalized by fixing the value of the GSH peak (whether [M + H]^+^ or [M – H]^−^) to 1.

### Detection of GSSG Using ^1^H NMR

The initial GSH concentration was fixed to 20 mM to provide enough sensitivity to be measured by ^1^H NMR. The catalyst concentration and temperature were adjusted to [Cu] = 20 mg L^–1^ and 25 °C for a total volume of 5 mL. The pH value was fixed to 7.4 using 100 mM of phosphate-buffered saline (PBS) solution. After 24 h of reaction, the sample was filtered and analyzed by ^1^H NMR. Water suppression in ^1^H NMR spectra was performed by using the Bruker Avance 400 MHz pulse program acquiring 32 scans for each sample. ^1^H NMR spectra were processed using MestreNova software.

### UV–Vis Analysis of the Reaction after DTNB Derivatization

The GSH concentration was measured using DTNB (Ellmann’s reagent) following previous protocols.^2^ The initial GSH concentration was fixed to 5 mM in order to mimic an intracellular ambient.^19^ The catalyst concentration and temperature were fixed at 0.05 mg mL^–1^ and 37 °C, respectively. Two pH conditions were evaluated for this reaction: 5.6 (fixed using CH_3_COONa/CH_3_COOH buffer 0.05 M) and 7.4 (using PBS 1× solution). The evolution of GSH concentration at different reaction times was monitored by tracking the absorbance at 412 nm by UV–vis spectroscopy, using a 2 mm optical path quartz cuvette. The GSH concentration in the reaction was analyzed by mixing 20 μL of the reaction mixture at the indicated times in Eppendorf tubes containing 3540 μL of TRIS (0.01 M) and 40 μL of DTNB (1 mg mL^–1^).

### Metal Analysis in Solution after the GSH Reaction

The concentrations of CuFeS_2_ NPs and GSH were kept at [CuFeS_2_] = 0.1 mg mL^–1^ and 5 mM, respectively. The pH was adjusted either to 5.6 using a 0.05 M CH_3_COOH/CH_3_COONa solution or 7.4 using PBS 1× solution. Both suspensions were stirred at 37 °C and constant agitation of 400 rpm. 200 μL was sampled at each time point (10 times up to 72 h) and centrifuged at 13,300 rpm for 10 min. Metal concentration in supernatants was analyzed by MP-AES. The remaining solid catalyst was analyzed by HRTEM, STEM-EDS, XRD, and DLS after the reaction.

### Identification of GsSH Oxidation Byproducts

1,3-Diphenylisobenzofuran (DPBF) was employed as a probe to measure the production of H_2_O_2_ during homogeneous GSH oxidation.^12^ 30 μL of 10 mM DPBF solution (in ethanol) was added to 2.5 mL of a mixture of ethanol/PBS (1×) (2:1). Catalyst and GSH concentration were 0.05 mg mL^–1^ and 5 mM, respectively. The absorbance measurement at λ = 411 nm of remaining DPBF by UV–vis spectroscopy was performed after centrifuging the sample (100 μL of reaction + 400 μL of ethanol/PBS 1× mixture) at 13,000 rpm for 5 min.

### Peroxidase (POD)-like Activity of CuFeS_2_

The oxidation of organic substrates using H_2_O_2_ was investigated with the colorimetric probe of TMB by monitoring the absorbance at λ = 652 nm. Different volumes of 200 mM H_2_O_2_ were added over a suspension containing 0.05 mg mL^–1^ catalyst and 0.1 mM TMB dissolved in 20 μL of DMSO. The pH of the reaction was maintained at 4.0, 5.6, or 6.5 using a 0.05 M CH_3_COOH/CH_3_COONa buffer solution. The experiment at pH = 7.4 was buffered using PBS 0.1 M. Thus, for UV–vis measurements the final concentrations in the reaction solution were 0.5, 1.0, 5.0, and 10.0 mM H_2_O_2_. The oxidation produced a blue color with a maximum absorbance centered at 652 nm.^20^

## Results and Discussion

### Synthesis and Characterization of the Chalcopyrite CuFeS_2_ Nanoparticles

**Figure 2:**
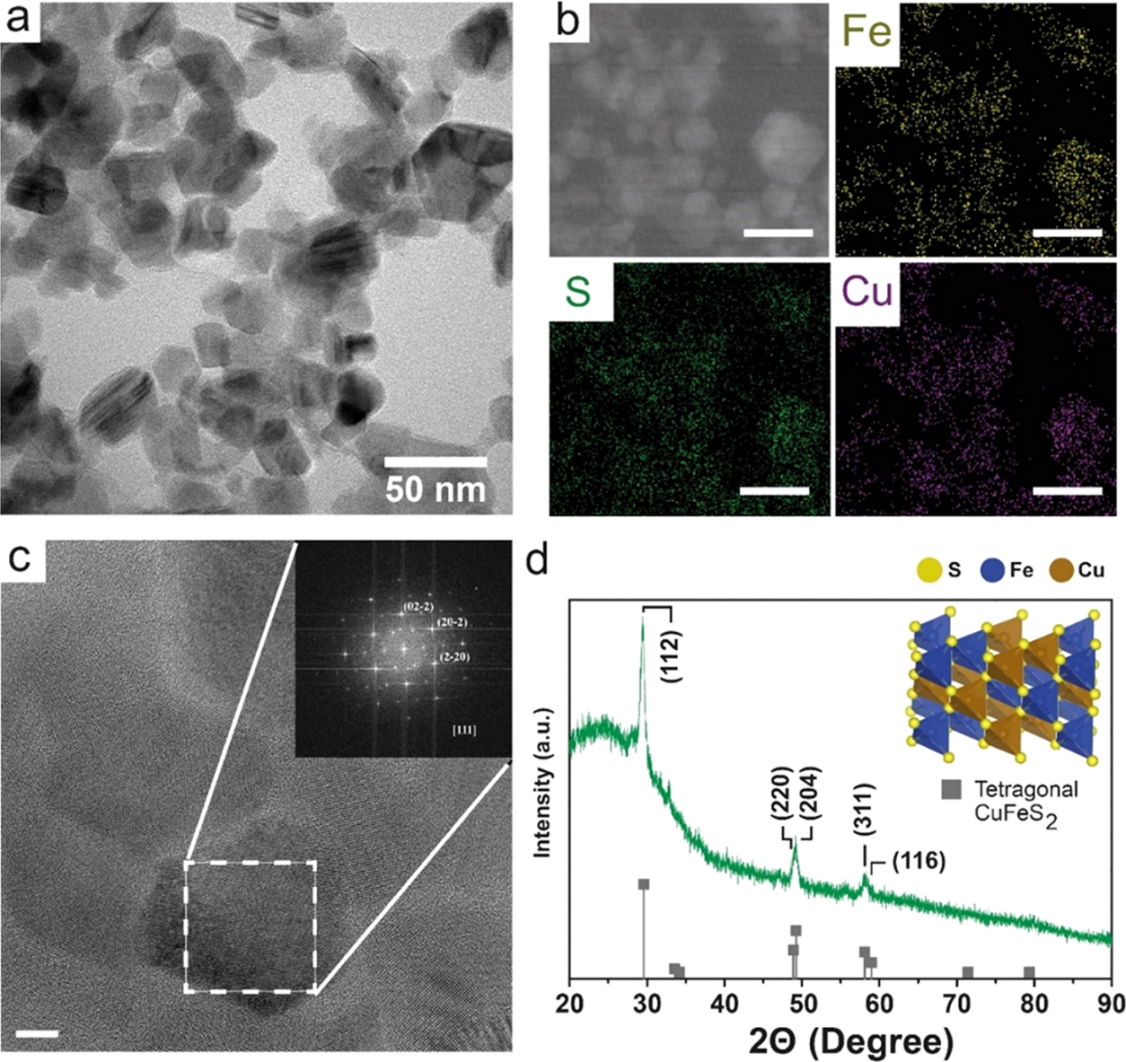
Physicochemical characterization of CuFeS_2_ nanoparticles: (a) low-magnification TEM images; (b) HAADF-STEM-EDS analysis of CuFeS_2_ NPs including elemental mapping of Fe, S, and Cu; scale bar = 50 nm; (c) HRTEM image of a single CuFeS_2_ nanoparticle and its FFT analysis where the spots have been indexed as the (022), (202), and (220) planes of the [111] zone axis of the tetragonal structure with cell parameters *a* = *b* = 5.27 and *c* = 5.194; scale bar = 5 nm; and (d) XRD pattern of CuFeS_2_ and standard pattern of JCPDS#00-035-0752 associated to the tetragonal crystalline structure of CuFeS_2_ (inset).

The synthesis of the CuFeS_2_ NPs used PVP as a capping agent to induce the growth of homogeneous particles and PEG as a coating agent in order to improve stability and biocompatibility. In addition, EG was used as cosolvent, hindering the agglomeration between particles formed at nearby nucleation points and thereby contributing to the final distribution of small, well-dispersed NPs.^2^ TEM images showed the formation of polyhedral morphologies with narrow diameter distribution (mean size of 35.7 ± 10.1 nm) (Figures 2a and S4). The hydrodynamic diameter of the particles in an aqueous solution at pH = 7.4 was determined by dynamic light scattering (DLS) to be 62 nm, exhibiting reasonable colloidal stability in water without forming aggregates larger than 200 nm (Figure S3). HAADF-STEM-EDS analysis of the sample revealed a homogeneous distribution of Fe, S, and Cu within the NPs (Figure 2b). HRTEM images and FFT analysis of localized areas revealed lattice spacing of 0.184, 0.185, and 0.188 nm, which correspond with the interplanar distances of (202), (022), and (220), respectively, of the zone axis [111] of the CuFeS_2_ tetragonal structure (Figure 2c). XRD confirmed the existence of this CuFeS_2_ tetragonal phase (JCPDS 00-035-0752)^21,22^ (Figure 2d).

**Figure 3:**
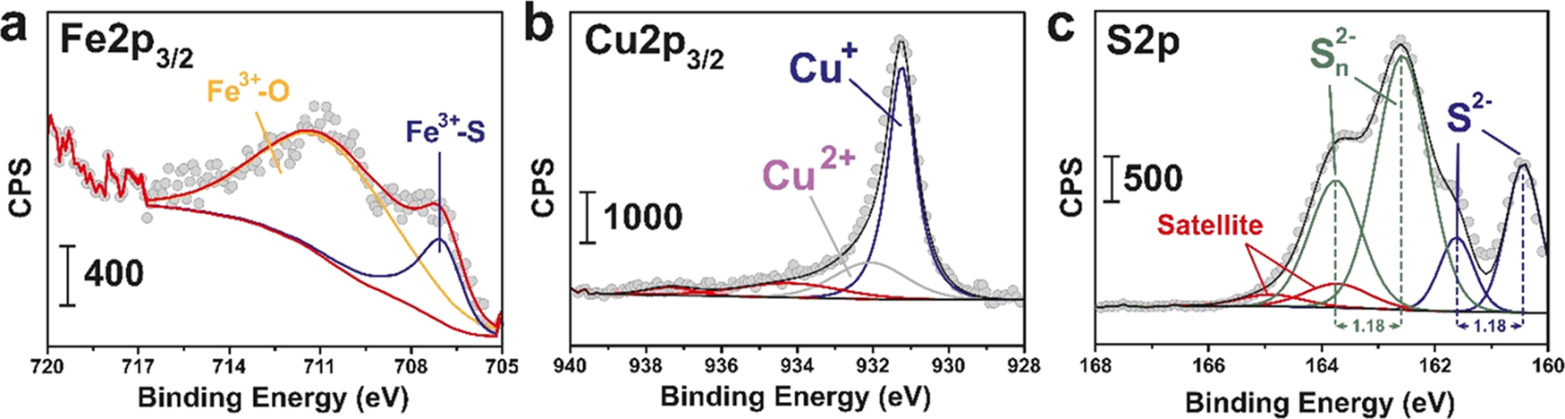
XPS characterization of CuFeS_2_ nanoparticles: (a) fitted XPS spectrum corresponding to the Fe2p_3/2_ region and exhibiting signals attributable to Fe^3+^–S and Fe^3+^–O species at 707.2 and 711.3 eV, respectively;^28,29^ (b) fitted XPS spectrum corresponding to the Cu2p_3/2_ region where Cu^+^ is the predominant oxidation state of Cu in CuFeS_2_; (c) analysis of the fitted XPS spectrum of the S 2p region evidencing the presence of polysulfide species in the surface, together with S^2–^ ions acting as linkers between metal atoms.

The characteristic Fourier transform infrared spectroscopy (FTIR) absorption peak of CuFeS_2_ is reported to be in the vicinity of 570 cm^–1^.^23^ In the case of PEG,^24^ most of the vibrations characteristic of C–O and C–C bonds appear at 840 and 1100 cm^–1^, respectively, while those characteristic of CH_2_ bonds are between 1145 and 1500 cm^–1^ (Figure S5). Raman spectra also confirmed the chalcopyrite structure (Figure S6) and showed peaks at 290, 411, and 499 cm^–1^ corresponding to the two pairs of S^2–^ vibrational modes^25,26^ and S–S stretching, respectively. Peaks at 81 and 229 cm^–1^ corresponded to the S^0^ lattice, which matches the Raman spectrum of the elemental S material. The peak at the larger Raman shift could be attributed to C-bond vibrations in this region.^27^ XPS fitting analysis corroborated the existence of the presence of the highest valence states for Fe(II) and Cu(I) catalytic species on the surface (Figure 3). X-ray photoemission peaks of the Fe2p_3/2_ region revealed a combination of Fe^3+^–S and Fe^3+^–O species,^28^ with peaks centered at 707.2 and 711.3 eV, respectively^29^ (Figure 3a). Likewise, the analysis of Cu 2p_3/2_ revealed the major presence of Cu^+^ species with binding energies of 931.3 eV with a small contribution of divalent Cu at 932.4 eV (Figure 3b) in good agreement with previous reports on chalcopyrite NPs.^25,26^ In the case of the S 2p region, three doublet contributions were successfully fitted. The main peaks centered at 160.4 and 162.5 eV peaks and separated by 1.18 eV, were attributed to S^2–^ and S*_n_*^2–^ species, respectively.^29^ S^2–^ is present within the crystal lattice acting as linkers between metals while we hypothesize that S*_n_*^2–^ species may remain at the surface due to the excess of S employed in the synthesis process (Figure 3c).

### CuFeS_2_ Catalytic Activity toward Heterogeneous-GSH Depletion

**Figure 4:**
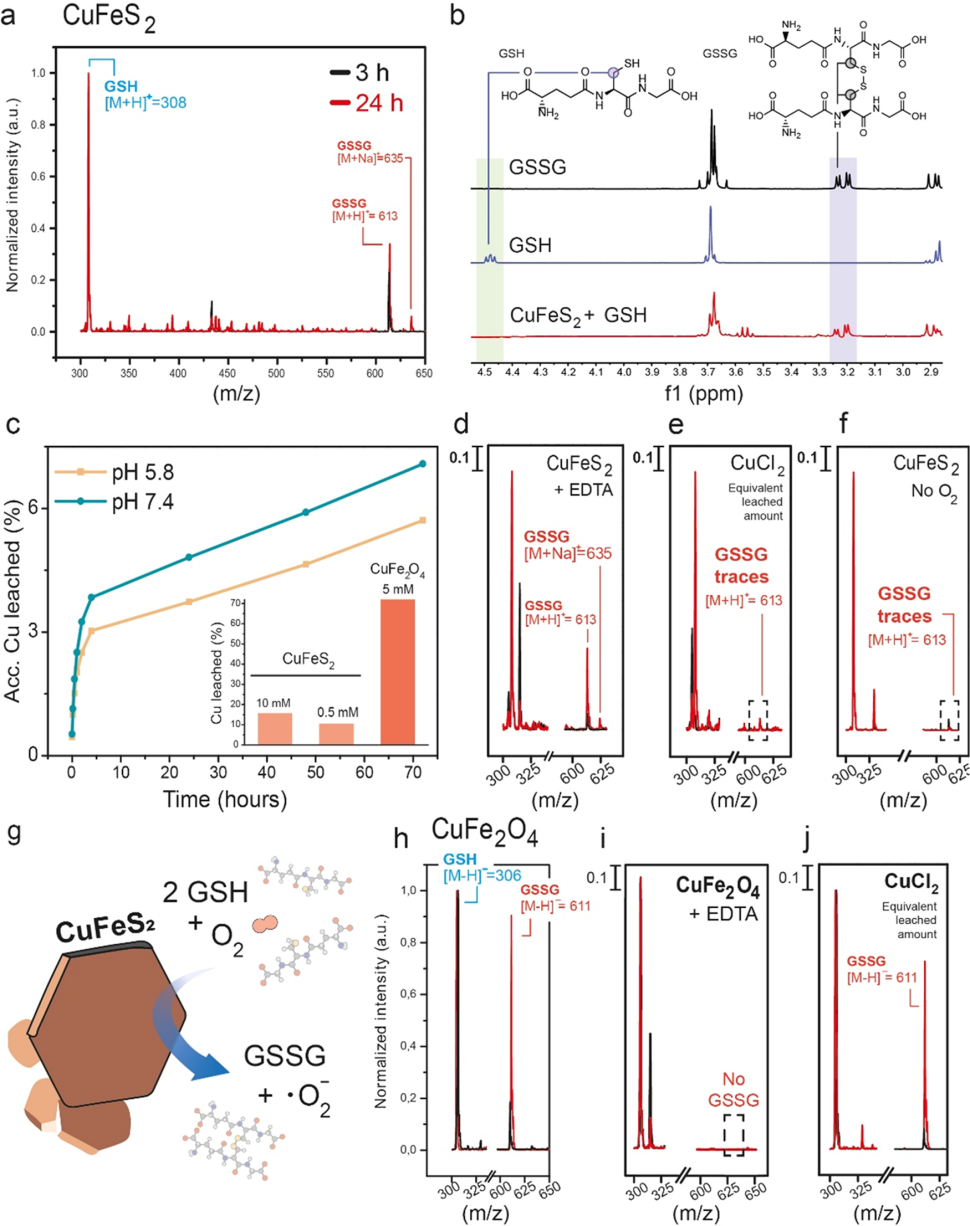
Heterogeneous GSH catalytic activity of CuFeS_2_ nanoplatelets. (a) Detection of GSSG ([M + H]^+^ = 613) via MS in the reaction mixture after 3 h (black line) and 24 h (red line); (b) ^1^H NMR spectra of commercial GSSG (top), GSH (middle) and CuFeS_2_ + GSH mixture after 24 h of reaction. Reaction conditions: [GSH]_0_ = 20 mM, [Cu] = 10 ppm, *T* = 25 °C, pH = 7.4 (phosphate buffer saline 50 mM); (c) metal analysis of the reaction supernatant at different reaction times both at pH = 5.8 (yellow line) and 7.4 (blue). Inset: percentage of released copper at different GSH concentrations in comparison with CuFe_2_O_4_ nanoparticles at 24 h, pH = 7.4; (d) detection of the reaction product GSSG ([M + H]^+^ = 613, [M + Na]^+^ = 635) by MS in the presence of 5 mM of EDTA to ensure all potentially released copper is instantly chelated; (e) MS analysis of the CuCl_2_ + GSH experiment after the addition of the equivalent amount of released copper from CuFeS_2_ after 24 h (0.032 mM); (f) MS spectra of the reaction after 3 h (black line) and 24 h (red line) where all dissolved O_2_ has been purged; (g) schematic illustration of the heterogeneous GSH oxidation onto CuFeS_2_ nanoplatelets to produce GSSG; (h) analysis of the reaction between GSH and copper releasing nanoparticles,^12^ CuFe_2_O_4_; (i) MS analysis of the reaction after the addition of 5 mM EDTA to chelate copper present in the solution; and (j) MS of the reaction in the presence of the equivalent amount of released copper in the form of CuCl_2_ (0.29 mM). All reaction conditions for all plots are listed below, except if specifically indicated: [GSH]_0_ = 5 mM, [Cu] = 10 ppm, *T* = 25 °C, pH = 7.4 (TRIS buffer 50 mM). MS spectra intensity was normalized to the intensity of GSH ion to a value of 1.

We investigated the reactivity of the CuFeS_2_/GSH system by using MS and ^1^H NMR to monitor both GSH and GSSG. The initial concentration of GSH was set to 5 mM in order to mimic the intracellular environment.^2,12,30^ We detected the product GSSG after 3 and 24 h of reaction by MS (detected ions: [M + H]^+^ = 613 and [M + Na]^+^ = 635) (Figure 4a). ^1^H NMR analysis also confirmed both the generation of GSSG and the consumption of GSH. Characteristic chemical shifts from GSSG (−SCH_2_–, 3.18 ppm) and GSH (−SCH_2_–, 4.45 ppm) increased and decreased, respectively (Figure 4b). We also monitored the GSH concentration during CuFeS_2_-assisted catalysis at early reaction times using Ellman’s reagent (DTNB) by UV–vis both at neutral and mildly acidic pH, characteristic of the TME (Figure S6). We found an absorbance at 412 nm decrease indicating the consumption of GSH with time at both pHs (Figure S6). In order to confirm whether this catalytic process was taking place via a heterogeneous pathway, we analyzed the metal content of the reaction supernatant via MP-AES (Figure 4c). To the best of our knowledge, this specific analysis to determine the presence of metals after GSH reaction and discern between homogeneous or heterogeneous catalysis using sulfur-based materials has not been reported in previous works available in the literature. Our results showed a cumulative release of 7.1% of the initial Cu after 72 h under physiological pH conditions and 5.7% under conditions of pH close to that of the TME (Figure 4c). Although the differences are not very significant, we tentatively attribute them to the major fraction of GSH containing deprotonated thiol groups at pH = 7.4. This makes the thiolate much more nucleophilic than the thiol group and more reactive toward transition metals such as Cu, thereby promoting its release from the particle.^2,12^ In contrast, Fe leaching levels from the CuFeS_2_ platform were much lower than for any of the explored conditions (Figure S7). We also studied copper leaching in the presence of 0.5 and 10 mM of GSH after 24 h of reaction at physiological pH without any significant changes. In contrast, an opposite trend was observed with the amount of released copper for the CuFe_2_O_4_ NPs (inset Figure 4c). This led us to think that a small fraction of oxidized CuFeS_2_ catalyst was susceptible to copper release in the presence of GSH. To evaluate whether this small amount of copper in solution could be responsible for the catalytic oxidation of GSH via a homogeneous pathway,^12^ we performed several control experiments. First, we carried out the reaction in the presence of EDTA, a well-known metal chelator with the aim of trapping any potential copper ions released and blocking the homogeneous pathway. In addition, we confirmed that EDTA did not induce a significant CuFeS_2_ lixiviation (Figure S8) and was able to prevent the GSH oxidation when using Cu^2+^ as a homogeneous catalyst (Figure S9). Although the GSSG product signal found after 3 h of reaction time was lower than that in the absence of EDTA, a significant amount of GSSG was formed after 24 h (Figure 4d). We suggest that this excess of EDTA can also be adsorbed onto the CuFeS_2_ surface blocking the catalytically active sites and slowing the reaction rates. In addition, we added the equivalent amount of released copper from CuFeS_2_ after 24 h in contact with 5 mM GSH to further confirm whether this Cu in solution was enough to significantly catalyze GSH oxidation in the homogeneous phase using CuCl_2_. We could not identify GSSG either after 3 or after 24 h of reaction (Figure 4e). We also evaluated the relevance of dissolved O_2_ in the reaction, as it is known to act as an electron acceptor in several organic oxidations.^31^ MS analysis of a suspension containing GSH and CuFeS_2_, where the O_2_ was previously purged, revealed the absence of newly formed GSSG (Figure 4f). Thus, we hypothesize that dissolved O_2_ can oxidize GSH once it is absorbed onto the catalyst surface. All of these results led us to identify this reaction as a heterogeneous catalytic process (Figure 4g). We performed the analogous experiments but in the presence of nanoparticles susceptible to release of copper in the presence of GSH, i.e., CuFe_2_O_4_.^12^ As expected, the combination of GSH and CuFe_2_O_4_ led to the appearance of GSSG ([M – H]^−^ = 611) (Figure 4h), but the presence of 5 mM EDTA completely stopped the reaction (Figure 4i). Furthermore, the addition of the equivalent amount of released copper using CuCl_2_ resulted in a large formation of GSSG (Figure 4j). These results confirmed that the methodology used to discern between homogeneous and heterogeneous processes was valid.

Only a scarce number of mixed Cu–Fe sulfide-based nanostructured analogues have reported GSH consumption.^32,33^ In the case of monometallic sulfides, CuS^34,35^ and FeS_2_^31^ were also reported to oxidize GSH. Tang et al. recently reported^34^ the synthesis of CuS NPs with the capability to oxidize GSH, but they attributed this oxidation to the Cu^2+^ released as a consequence of a combination of the lower pH in tumors and near-infrared (NIR) irradiation. Meng et al.^31^ designed FeS_2_ NPs able to catalyze GSH oxidation at pH = 4.5 using O_2_ as an electron acceptor to yield H_2_O_2_ as a byproduct. Also, the homogeneous catalytic GSH oxidation reaction has been claimed to take place using Cu^2+^ cations, either present in the aqueous media^36^ or released from the mixed oxide catalyst CuFe_2_O_4_,^12^ as catalysts to yield H_2_O_2_ and ^•^O_2_^–^ species. We then probed the byproduct generated in the reaction with GSH using 1,3-diphenylisobenzofuran (DPBF). Prior to studying the CuFeS_2_–GSH reaction, we evaluated whether DPBF was capable of reacting with H_2_O_2_ or with ^•^O_2_^–^, two potential byproducts of the GSH oxidation reaction.^12,31^ Our results suggested that DPBF could not react with added H_2_O_2_ either in the absence or presence of GSH (Figure S10a,b), but did with KO_2_, a source of superoxide anions (Figure S10c,d). Therefore, a decrease in DPBF absorbance present in a mixture of CuFeS_2_ with GSH can be related to the generation of ^•^O_2_^–^ (Figure S11). We indirectly detected ^•^O_2_^–^ in the presence of 5 mM GSH using CuFeS_2_ as a catalyst both in acidic and neutral pH (Figure S12) as the DPBF absorbance at 411 nm peak decreased with reaction time without a significant Cu (Figure 4c) or Fe release (Figure S13).

Given the potential ability of CuFeS_2_ toward impairing the redox homeostasis by depleting GSH levels while raising ROS concentrations, we decided to investigate its interaction with an abundant molecule present in the TME, H_2_O_2_.^4,37^ This scenario can be leveraged by a multitude of transition metal-based nanocatalysts using H_2_O_2_ to oxidize biomolecules within the cell and increase the oxidative stress, leading to apoptosis.^8^ The capability of certain nanomaterials of oxidizing organic substrates using H_2_O_2_ is known as peroxidase-like (POD) activity and occurs preferentially at pH 3.5–4.5 for iron oxide nanocatalysts.^8,20^ In fact, the simultaneous presence of Cu and Fe within the same crystalline lattice enhanced the Fenton activity at a relatively higher pH value in comparison with the results reported by Meng et al.,^31^ which were carried out at pH = 4.5 using a FeS_2_ catalyst. This trend has been previously observed by Chen et al.^38^ and Wang et al.^39^ in a wide range of pH (7.4–5.6) with other CuFe–S nanocatalysts reporting analogous ^•^OH production. Then, we evaluated the POD-like activity of CuFeS_2_ using TMB as the organic substrate at different pH values and monitoring the absorbance of the produced oxidized TMB by UV–vis spectroscopy at different pH values (Figure S13a). The highest activity was found at pH = 4 (Figure S13b) in agreement with the typical trend displayed by transition metal-based POD-like nanomaterials.^8,20^ This acidic pH is within the range of the value reported for lysosomes,^40^ one the subcellular localization where nanoparticles are mostly accumulated after internalization,^41^ including metal sulfides.^31^ POD-like activity of CuFeS_2_ was reduced while increasing pH value (Figure S13c,d) until it is completely canceled at pH 7.4 (Figure S13e), which can help to prevent further damage to healthy tissues. In addition, given the reactivity of CuFeS_2_ + H_2_O_2_ toward TMB at pH 4, we decided to check if the presence of GSH could generate additional H_2_O_2_. After 24 h of reaction, CuFeS_2_ produced oxidized TMB both in the absence of GSH. We suggest that dissolved molecular O_2_ can be activated by the CuFeS_2_ surface in a similar way as in the case of noble-metal nanozymes^42^ or FeS.^31^ However, the signal was not much larger than the blank experiment, so we expect this process to be more unfavorable than GSH oxidation. The presence of GSH did not increase the oxidized TMB signal, so H_2_O_2_ does not seem to be produced during GSH oxidation (Figure S14).

We further evaluated the spent nanocatalyst after the reaction. Microscopy analysis after the reaction with 5 mM of GSH in physiological conditions revealed no significant differences in terms of crystallinity and composition in comparison with starting nanocatalyst, thereby confirming the negligible release of metals from the CuFeS_2_ NPs (Figure S15a) or any significant variation in their chemical composition (Figure S15b). Hence, the XRD spectrum of CuFeS_2_ after the reaction presented intact the peaks corresponding to (112), (220), (204), (311), and (116) planes (Figure S16).^21,22^ Finally, we also measured the hydrodynamic diameter of the particle by DLS without finding significant differences from the original sample (Figure S17). All of these data pointed out how the structural integrity of the CuFeS_2_ catalyst was intact during the reaction with GSH, acting as a heterogeneous catalyst.

## Conclusions

Copper–iron-based chalcogenides can be a promising alternative to their mixed oxide counterparts to minimize the uncontrolled release of Cu species under biological conditions that somehow limits their potential translation into in vivo applications. The co-existence of Cu and Fe in chalcopyrite nanoparticles takes advantage of the synergetic action of both transition metals to boost the Fenton-like activity via a charge transfer mechanism from Cu^+^ to Fe^3+^ to regenerate the active Fe^2+^.^15^ The CuFes_2_ nanocatalyst exhibits similar GSH oxidation capability to the Cu–Fe oxides both at pH = 5.6 and 7.4 without incurring a significant lixiviation. Moreover, we could detect ^•^O_2_^–^ as a main byproduct of the GSH oxidation. Then, CuFeS_2_ has the potential to disrupt the redox equilibrium in cells by depleting antioxidant species while rising ROS levels. Additionally, CuFeS_2_ can further activate intracellular H_2_O_2_ to oxidize organic substrates in acidic environments such as lysosomes. In this way, the CuFeS_2_ catalyst represents a potential and promising candidate to trigger all necessary reactions to perform efficient CDT in a single platform, without the requirement of homogeneous catalysis and the concomitant loss of Cu that partially hinders the application of oxide-based nanoparticles for cancer therapy.

## Supporting Information Available

Synthesis route to CuFeS_2_ nanoparticles; representative TEM images of CuFeS_2_ nanoplates; hydrodynamic diameter of CuFeS_2_ nanoparticles; FTIR analysis of CuFeS_2_ nanoparticles; Raman spectrum of CuFeS_2_–PEG nanoparticles; UV–vis spectra monitoring the evolution of TNB^2–^ as an indirect reactant to detect and quantify GSH consumption; released Fe from CuFeS_2_ nanoplatelets; evolution of the Cu and Fe metals leached from CuFeS_2_; MS analysis of the reaction of CuCl_2_ + GSH with a 5 mM EDTA concentration; DPBF reactivity toward H_2_O_2_ or KO_2_; detection of H_2_O_2_ as a byproduct of heterogeneous GSH oxidation using DPBF; UV–vis spectra of DPBF at different times in GSH catalysis at different pH values; peroxidase (POD)-like activity of CuFeS_2_ nanoparticles; UV–vis spectra of oxidized TMB in different mixtures; CuFeS_2_ analysis after reaction with 5 mM GSH; XRD analysis of CuFeS_2_ after 24 h in the presence of 5 mM GSH, and DLS analysis of a CuFeS_2_ sample after reaction with 5 mM GSH (PDF)
