# Supplementary material for: Heterogeneous-Driven Glutathione Oxidation: Defining the Catalytic Role of Chalcopyrite Nanoparticles
Source: J Phys Chem C Nanomater Interfaces. 2023 Jul 12;127(29):14146–54. doi: 10.1021/acs.jpcc.3c00987 (PMC10388351; doi:10.1021/acs.jpcc.3c00987)
Supplement: Supplementary file 1 — jp3c00987_si_001.pdf [file jp3c00987_si_001.pdf]

Supporting Information

Heterogeneous-driven Glutathione Oxidation: defining the Catalytic Role of Chalcopyrite Nanoparticles

Leticia Sanchez-Uriel[†,§,‡,⁑], *Javier Bonet-Aleta*[†,§,‡,⁑,*], *Alfonso Ibarra and Jose L. Hueso*[†,§,‡,#,*]

Leticia Sanchez-Uriel[†,§,‡,⁑], *Javier Bonet-Aleta*[†,§,‡,⁑,*], *Alfonso Ibarra and Jose L. Hueso*[†,§,‡,#,*]

[†]Instituto de Nanociencia y Materiales de Aragon (INMA) CSIC-Universidad de Zaragoza, Campus Rio Ebro, Edificio I+D, C/ Poeta Mariano Esquillor, s/n, 50018, Zaragoza, (Spain).

[§]Networking Res. Center in Biomaterials, Bioengineering and Nanomedicine (CIBER-BBN), Instituto de Salud Carlos III; 28029 Madrid (Spain)

‡Department of Chemical and Environmental Engineering, University of Zaragoza, Campus Rio Ebro, C/María de Luna, 3, 50018 Zaragoza (Spain).

Laboratorio de Microscopias Avanzadas (LMA), Universidad de Zaragoza, Zaragoza 50018, Spain

#Instituto de Investigación Sanitaria (IIS) de Aragón, Avenida San Juan Bosco, 13, 50009 Zaragoza, (Spain).

‡ These authors contributed equally

*Corresponding authors: jbaleta@unizar.es; jlhueso@unizar.es

SUPPORTING INFORMATION INDEX

Figure S1 . Synthesis route of $CuFeS_2$ nanoparticles. ...3

Figure S2. TEM images of $CuFeS_2$ nanoplates ...4

Figure S3. Hydrodynamic diameter of $CuFeS_2$ nanoparticles. ...5

Figure S4. FTIR analysis of $CuFeS_2$ nanoparticles. ...6

Figure S5. Raman spectra of $CuFeS_2$. ...7

Figure S6. Analysis of GSH levels by DTNB assay. ...8

Figure S7. Released Fe from $CuFeS_2$ nanoplatelets during catalysis ...9

Figure S8. Released metals from $CuFeS_2$ in the presence of EDTA) 10

Figure S9. MS analysis of the reaction of $CuCl_2$+GSH with a 5 mM EDTA 11

Figure S10. DPBF reactivity towards H_2O_2 or KO_2. 12

Figure S11. Detection of $\cdot O_2^-$ as by-product 13

Figure S12. UV-vis spectra of DPBF at different times...14

Figure S13. Peroxidase-like activity of $CuFeS_2$ 15

Figure S14. Analysis of oxidized TMB in the presence of GSH 16

Figure S15. $CuFeS_2$ analysis after reaction with 5 mM GSH 17

Figure S16. XRD analysis of $CuFeS_2$ after 24 h in the presence of 5 mM GSH at pH = 7.4. 18

Figure S17. DLS analysis of a $CuFeS_2$ sample after reaction with 5 mM GSH at pH = 7.4 revealed no significant differences to the original hydrodynamic diameter. 19

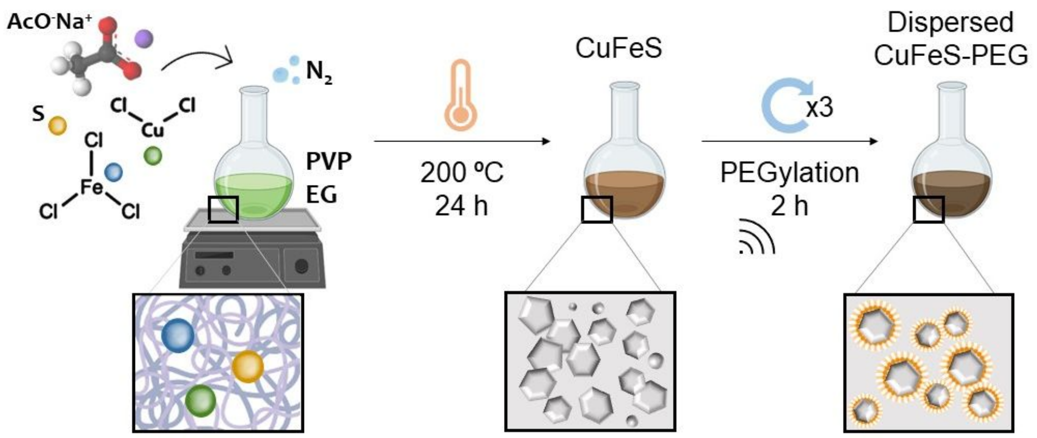

Figure S1. Synthesis route of $CuFeS_2$ nanoparticles. PVP, acting as capping agent was firstly dissolved in Ethylene Glycol (EG), followed by the addition of metal precursors ($FeCl_3$ and

$CuCl_2$), CH_3COONa and elemental S. After growth-phase at 200 ºC for 24 h, the NPs were PEGylated to enhance its dispersion and biocompatibility. Finally, the NPs were washed with EtOH:H_2O and H_2O, in different cycles.

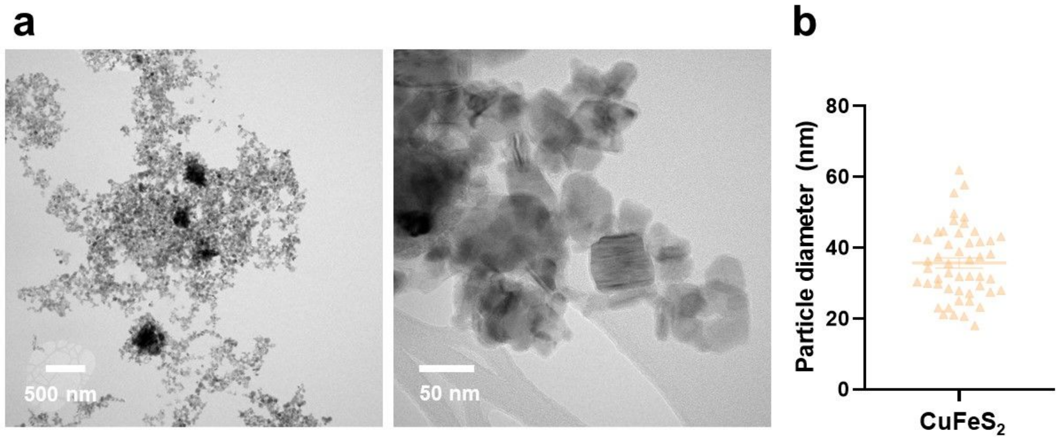

Figure S2. (a) Representative TEM images of $CuFeS_2$ nanoplates at different scales and (b) size distribution

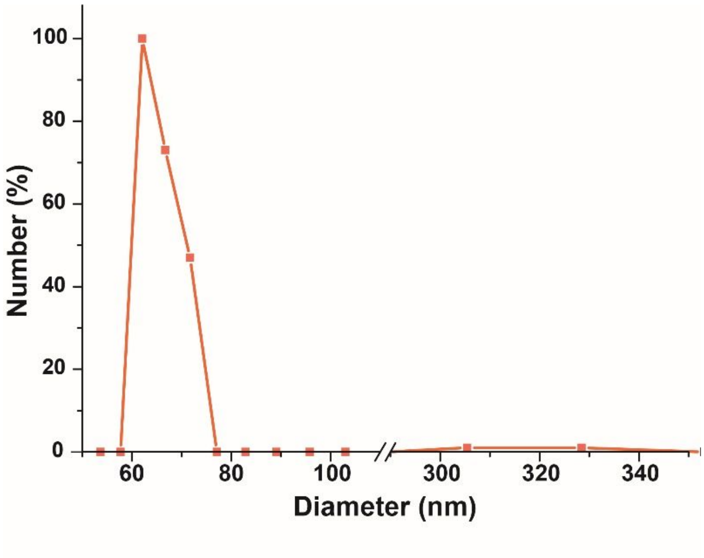

Figure S3. Hydrodynamic diameter of $CuFeS_2$ nanoparticles in aqueous solution at pH = 7.4.

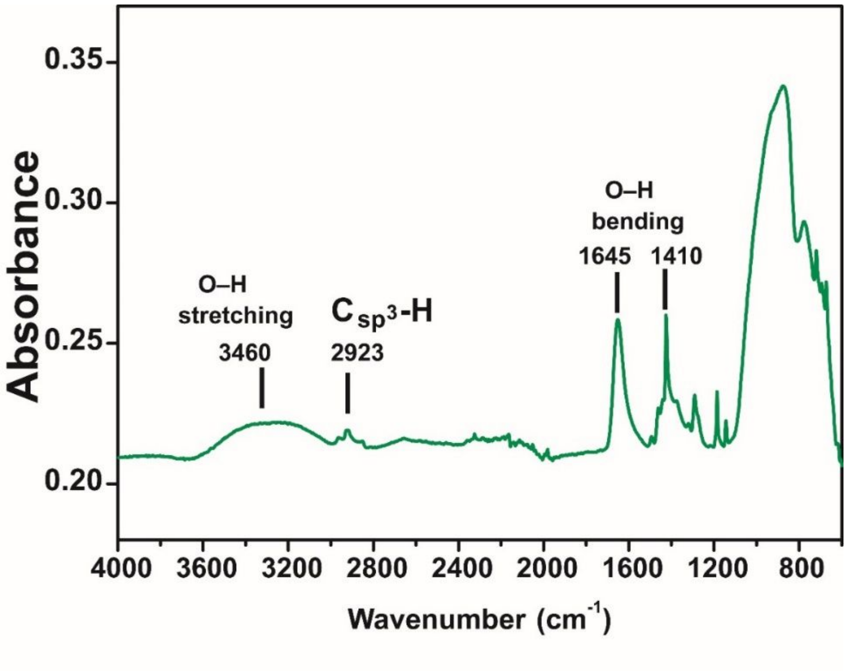

Figure S4. FTIR analysis of $CuFeS_2$ nanoparticles.

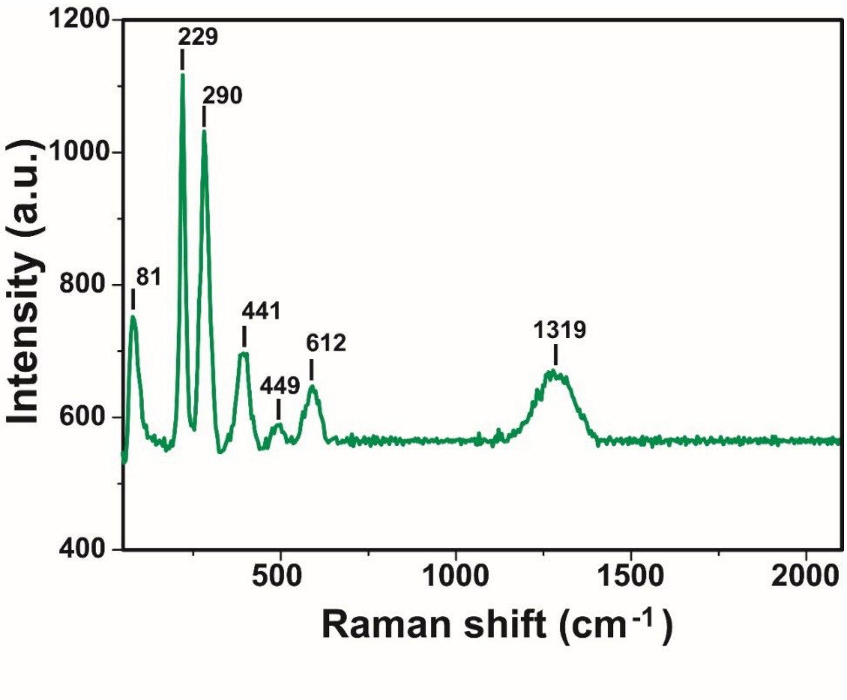

Figure S5. Raman spectrum of $CuFeS_2$-PEG nanoparticles. Peaks appearing at 290, 411 and 499 cm^{-1} correspond to the presence of S^{2-} and S-S species, respectively, while peaks at 81 and 229 cm^{-1} can be attributed to the presence of a small amount of S_0 lattice.

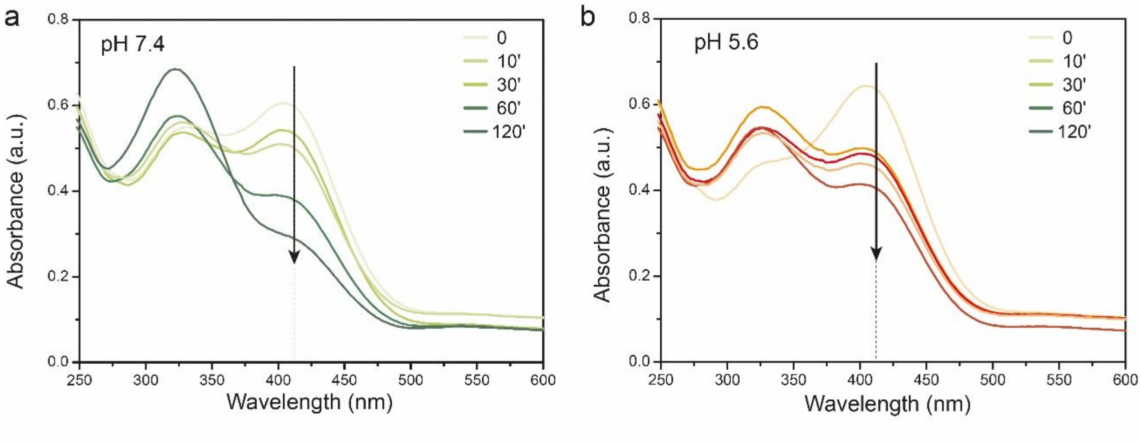

Figure S6. UV-vis spectra monitoring the evolution of TNB^{2-} as an indirect reactant to detect and quantify GSH consumption: (a) experiments carried out at pH = 7.4 (adjusted with PBS 1X) ; (b) experiments performed at pH = 5.6 (adjusted with CH_3COOH/CH_3COONa 0.05 M). Reaction conditions: $[CuFeS_2]$ = 0.05 mg·mL^{-1}, $[GSH]_0$ = 5 mM, T = 37 ºC.

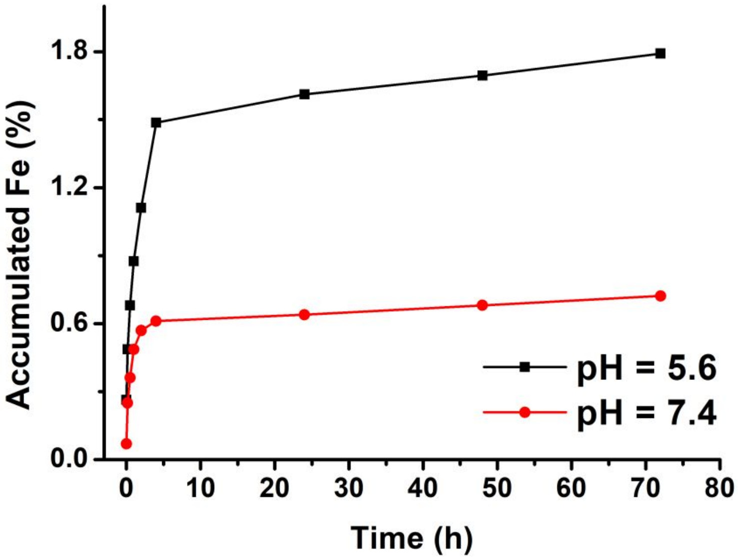

Figure S7. Released Fe from $CuFeS_2$ nanoplatelets at different pH in the presence of 5 mM of GSH at pH = 5.6 (black squares) and pH = 7.4 (red circles). After 72 h, accumulated Fe release is below 2%, which makes the potential homogeneous process negligible in comparison to heterogeneous-driven catalysis.

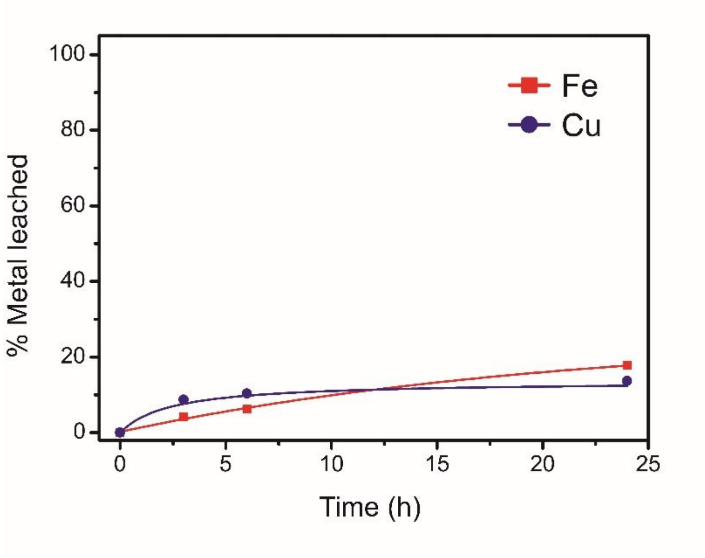

Figure S8. Evolution of the copper (circles) and iron (squares) metals leached from $CuFeS_2$ in the presence of 5 mM of EDTA after 24 h. Reaction conditions: 37ºC, pH = 7.4 (buffer TRIS 0.01 M).

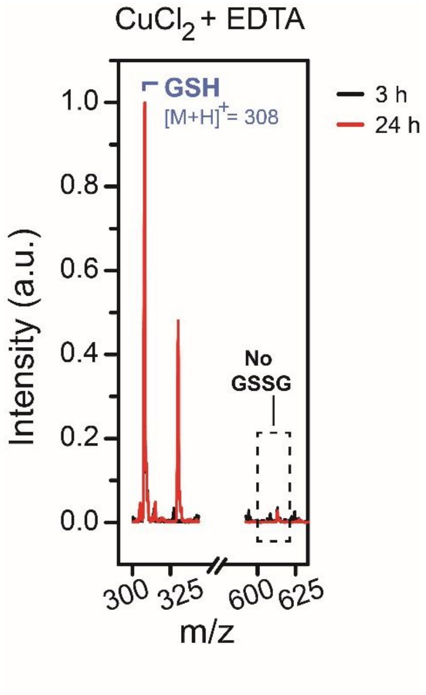

Figure S9. MS analysis of the reaction of $CuCl_2$+GSH with a 5 mM EDTA concentration in the reaction revealing no GSSG formation. Reaction conditions: $[GSH]_0$ = 5 mM, $[CuCl_2]$ = 0.29 mM, [EDTA] = 5 mM, T = 37 ºC, pH = 7.4 (buffered using TRIS 0.01 M).

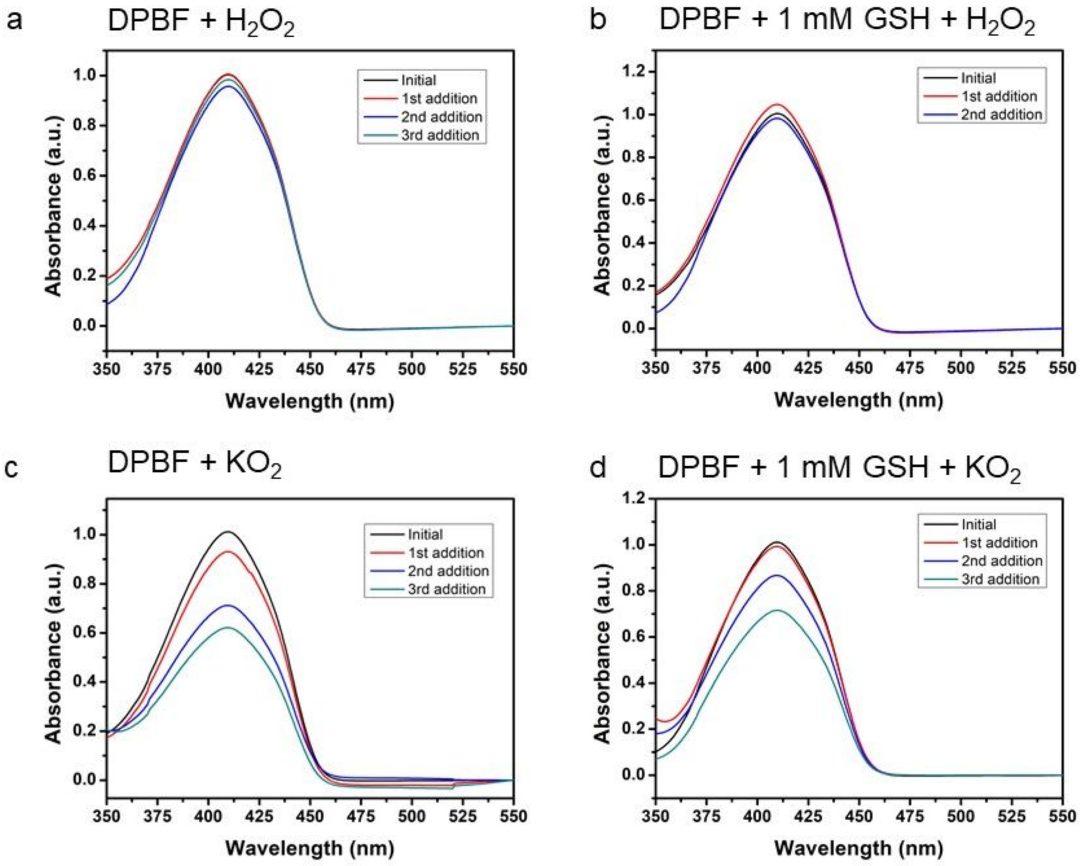

Figure S10. DPBF reactivity towards H_2O_2 or KO_2. UV-vis spectra of DPBF after the addition of several equivalents of H_2O_2 in the absence (a) or in the presence (b) of 1 mM GSH; Evolution of DPBF UV-vis spectra after the addition of a superoxide source, KO_2, (c) in the absence or (d) in the presence of 1 mM GSH.

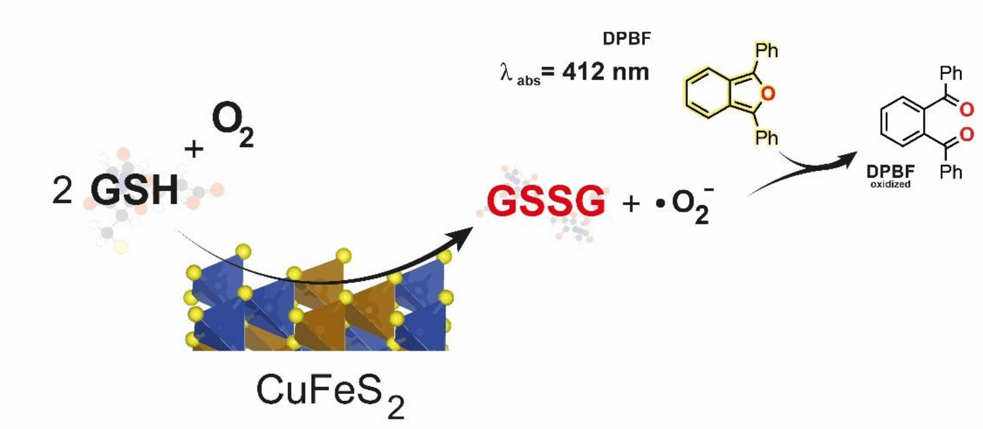

Figure S11. Detection of H_2O_2 as by-product of heterogeneous GSH oxidation using DPBF. Oxidation of GSH by $CuFeS_2$ produces H_2O_2 as by-product, as dissolved O_2 can act as electron acceptor. Generated $•O_2^-$ can further react with DPBF probe, decreasing the absorbance at 412 nm.

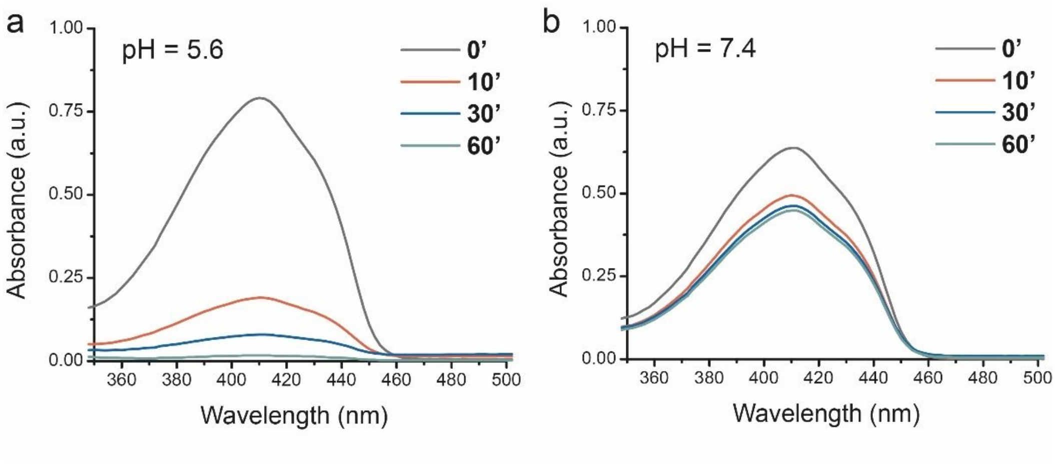

Figure S12. UV-vis spectra of DPBF at different times in GSH catalysis at different pHs (a) 5.6 and (b) 7.4 consequence of the formation of H_2O_2 during the oxidation process of GSH. Reaction conditions: $[CuFeS_2]$ = 0.1 mg·mL^{-1}, $[GSH]_0$ = 5 mM, $[DPBF]_0$ = 0.12 mM.

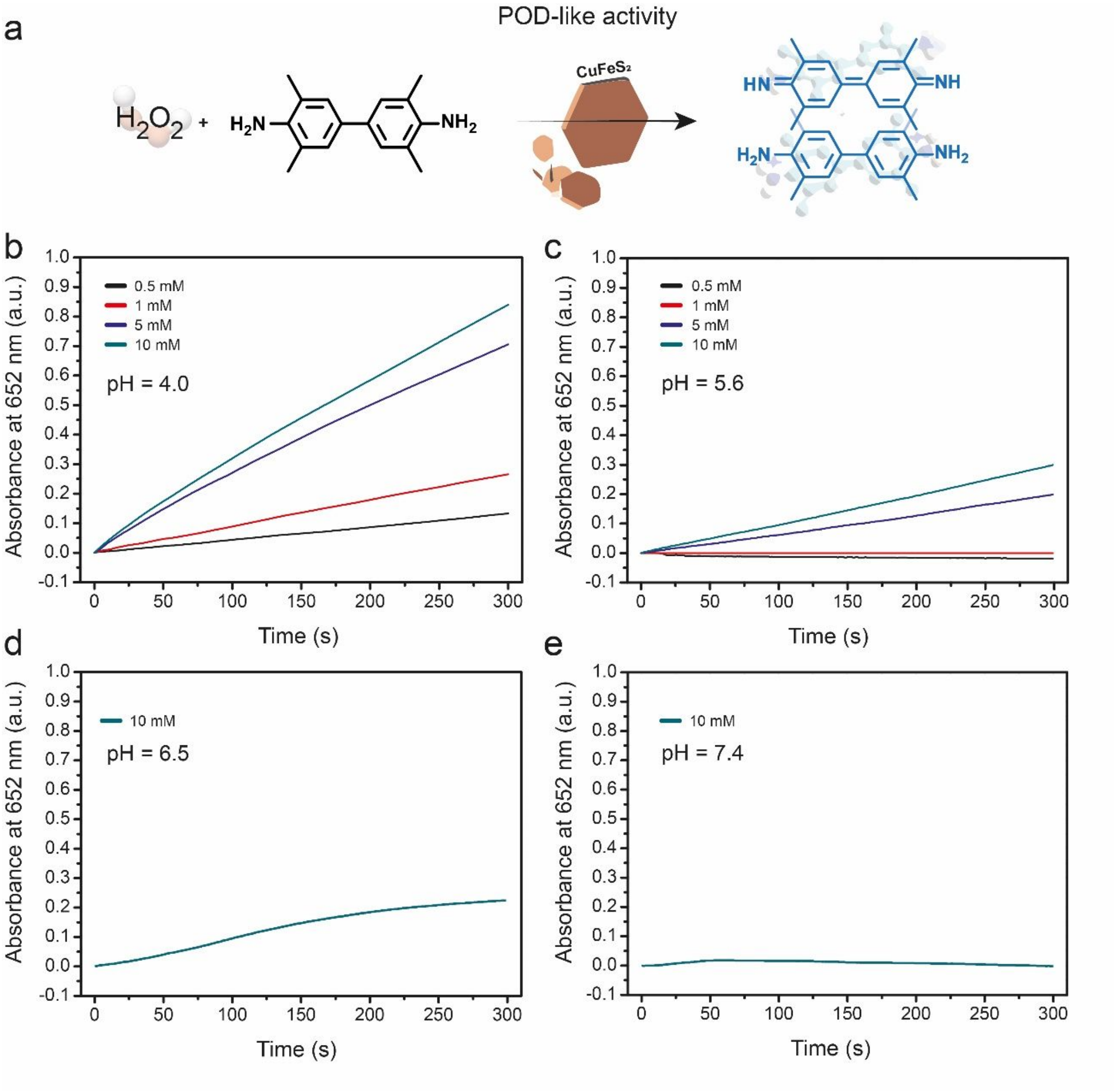

Figure S13. **(a)** Schematic representation of the Peroxidase (POD)-like activity of $CuFeS_2$ nanoparticles. The catalytic oxidation of an organic substrate as TMB using H_2O_2 yields a blue-colored product, TMB_{ox} which can be monitored through its absorbance at 652 nm; **(b-e)** Evolution of the absorbance at 652 nm produced by TMB_{ox} after the addition of different H_2O_2 concentrations confirmed the POD-like activity of $CuFeS_2$ nanoplatelets. Reaction conditions: [Cu] = 0.864 ppm, $[TMB]_0$ = 1 µM, T = 25 ºC. pH = 4.0, 5.6 and 6.5 was adjusted with CH_3COOH/CH_3COONa 0.05 M. For pH = 7.4, the buffer employed was PBS 0.1 M.

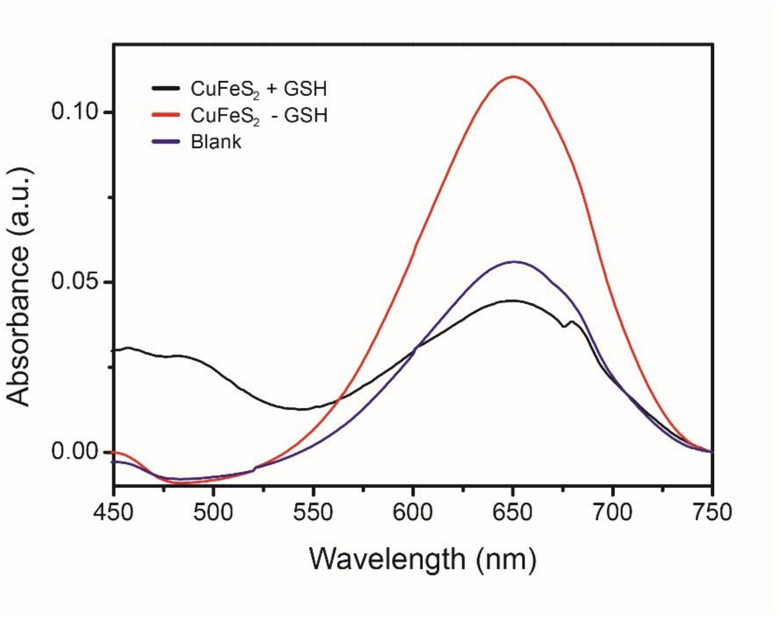

Figure S14. UV-vis spectra of oxidized TMB in different mixtures: $CuFeS_2$ + GSH (black line), $CuFeS_2$ without GSH (red line) and blank (blue line) after 24 h of incubation at room temperature. Reaction conditions: [Cu] = 0.864 ppm, $[TMB]_0$ = 1 μM, $[GSH]_0$ = 1 mM T = 25 ºC.

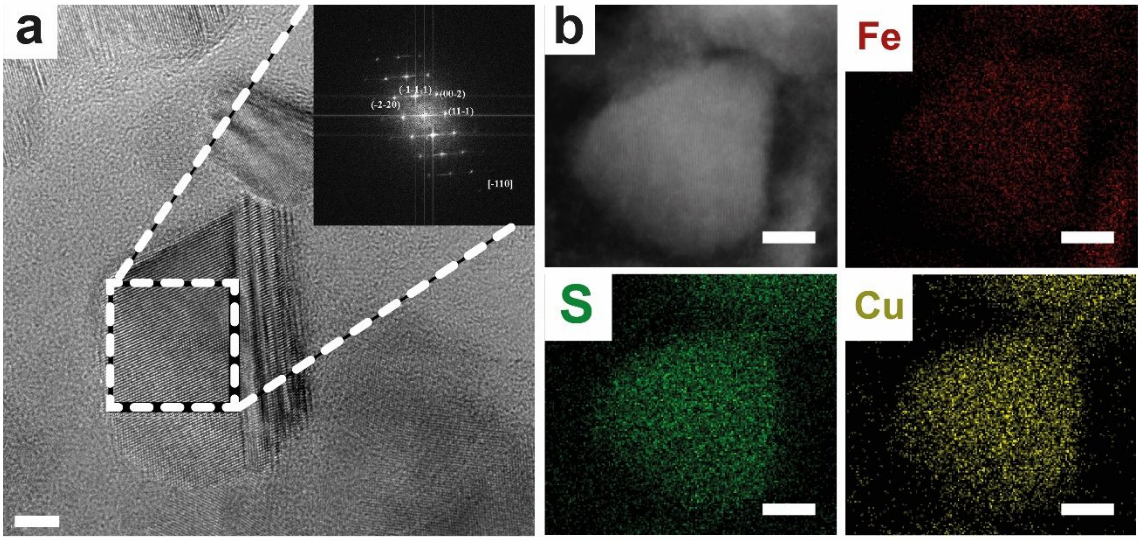

Figure S15. $CuFeS_2$ analysis after reaction with 5 mM GSH (a) HRTEM image of a single $CuFeS_2$ particle; inset: FFT analysis of planes. The presence of crystalline planes after reaction with GSH demonstrates that the catalyst remains intact even though being capable of catalyze oxidation. Scale bar: 10 nm; (b) STEM-EDS analysis of a single particle reveals a homogeneous distribution of Fe, Cu and S after reaction. Scale bar: 5 nm.

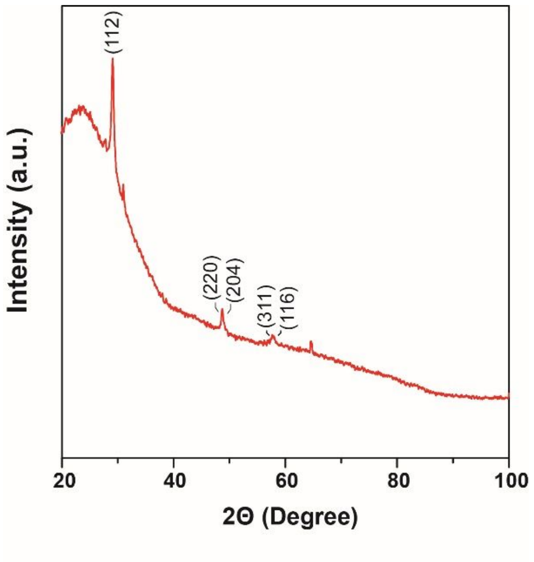

Figure S16. XRD analysis of $CuFeS_2$ after 24 h in the presence of 5 mM GSH at pH = 7.4.

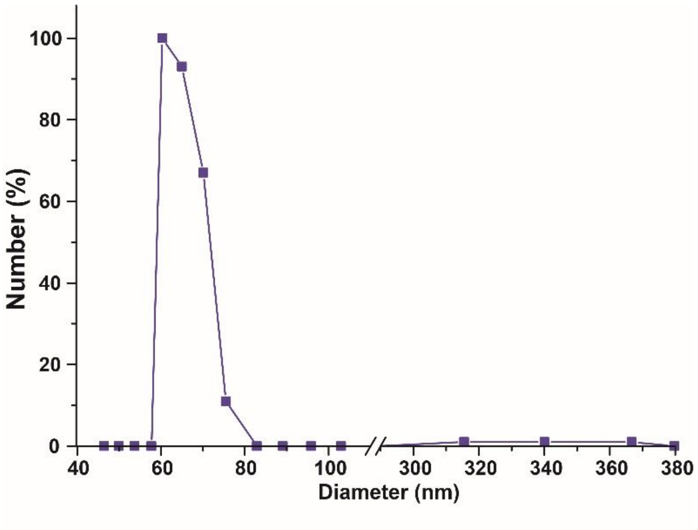

Figure S17. DLS analysis of a $CuFeS_2$ sample after reaction with 5 mM GSH at pH = 7.4 revealed no significant differences to the original hydrodynamic diameter.

SUPPORTING REFERENCES

1. Bonet-Aleta, J.; Encinas-Gimenez, M.; Urriolabeitia, E.; Martin-Duque, P.; Hueso, J. L.; Santamaria, J., Unveiling the interplay between homogeneous and heterogeneous catalytic mechanisms in copper–iron nanoparticles working under chemically relevant tumour conditions. Chemical Science 2022, 13 (28), 8307-8320.
